# Half-life time prediction of developing first-line antiretroviral treatment failure and its risk factors among TB and HIV co-infected children in Northwest Ethiopia; multi setting historical follow-up study

**DOI:** 10.1186/s12887-022-03177-6

**Published:** 2022-03-03

**Authors:** Ermias Sisay Chanie, Achenef Asmamaw Muche, Mengistu Berhanu Gobeza, Eshetie Molla Alemu, Wondimnew Desalegn Addis, Melkalem Mamuye Azanaw, Alemayehu Digssie Gebremariam, Desalegn Tesfa, Melaku Tadege Engidaw, Getaneh Atikilit, Sofonyas AbebawTiruneh, Getachew Arage

**Affiliations:** 1 Department of Paediatrics and Child Health Nursing, College of Health Sciences, Debre Tabor University, Debre Tabor, Ethiopia; 2 Department of Epidemiology and Biostatistics, University of Gondar, Gondar, Ethiopia; 3 Department of Paediatrics and Child Health Nursing, College of Medicine Health Sciences, University of Gondar, Gondar, Ethiopia; 4 Department of Public Health, College of Health Sciences, Debre Tabor University, Debre Tabor, Ethiopia

**Keywords:** Half-life, Treatment failure, TB and HIV co-infected, Children, Ethiopia

## Abstract

**Background:**

Even though treatment failure is higher among TB and HIV infected children in a resource-limited setting, there is no prior evidence in general and in the study area in particular. Hence, this study was aimed at determining the half-life time prediction of developing first-line antiretroviral treatment failure and its risk factors among TB and HIV co-infected children.

**Methods:**

A historical follow-up study was employed among 239 TB and HIV co-infected children from January 2010-December 2020. The data was entered into Epi data version 4.2.2 and exported to STATA 14.0 Software for analysis. The Kaplan-Meier plot was used to estimate the half-life time to develop treatment failure. The required assumption was fulfilled for each predictor variable. Additionally, those variables having a *p*-value ≤0.25 in the bivariable analysis were fitted into a multivariable Cox-proportional hazards regression model. *P*-value, < 0.05 was used to declare a significant association.

**Results:**

A total of 239 TB and HIV co-infected children were involved in this study. The overall half-life time to develop first treatment failure was found to be 101 months, with a total of 1027.8 years’ follow-up period. The incidence rate and proportion of developing first-line treatment failure were 5.5 per 100 PPY (Person-Year) [CI (confidence interval): 3.7, 6.9] 100 PPY and 23.8% (CI; 18.8, 29.7) respectively. Factors such as hemoglobin 10 mg/dl [AHR (Adjusted Hazard Ratio): 3.2 (95% CI: 1.30, 7.73), severe acute malnutrition [AHR: 3.8 (95% CI: 1.51, 79.65), World Health Organization stage IV [AHR: 2.4 (95% CI: 1.15, 4.93)], and cotrimoxazole prophylaxis non user [AHR: 2.3 (95% CI: 1.14, 4.47)] were found to be a risk factor to develop treatment failure.

**Conclusion:**

In this study, the half-life time to develop first-line treatment failure was found to be very low. In addition, the incidence was found to be very high. The presence of hemoglobin 10 mg/dl, severe acute malnutrition, World Health Organization stage, and non-use of cotrimoxazole prophylaxis were discovered to be risk factors for treatment failure. Further prospective cohort and qualitative studies should be conducted to improve the quality of care in paediatric ART clinics to reduce the incidence or burden of first line treatment failure among TB and HIV co-infected children.

## Background

There has been an increase in the use of anti-retroviral therapy (ART) services worldwide, particularly in resource-limited settings [1, 2], including Ethiopia [3, 4]. However, treatment failure poses substantial challenges for care, particularly among children who live with a scarcity of monitoring tools for evaluating the response to ART [3–5]. Although the World Health Organization stage (WHO) suggested regular monitoring and evaluating viral load is preferred to notice treatment failure due to its good specificity and sensitivity, clinical and immunological criteria are widely used in Ethiopia due to financial constraints [3, 4].

ART has health benefits for Human Immune Deficiency Virus (HIV) infected children by reducing the progress of the infection and chances of transmission to others [6, 7]. However, its benefits are markedly compromised due to treatment failure [8]. Retaining HIV-infected children on ART for an extended period of time without treatment failure is required to achieve maximum efficacy, minimize toxicity, and prevent the development of drug resistance [1].

Prevention of treatment failure is crucial in the context of lifetime treatment and limited drug availability [9]. However, treatment failure is usually seen in HIV infected children in a resource-limited setting, which decreases survival [9, 10]. Children who have had treatment failure may develop a variety of opportunistic infections and may develop drug resistance [11]. The development of drug-resistant virus strains can be an extra burden if this virus transmits to others [12]. Furthermore, the emergence of drug resistance can increase the cost and tolerability of second-line ART regimens [12]. Furthermore, the emergence of drug resistance can increase the cost and tolerability of second-line ART regimens [2].

Prevention and management of twin epidemics HIV and Tuberculosis (TB) co-infection and its associated complications are on the ART program’s national agenda [13–15], but treatment failure continues to reduce the quality of care and reduce survival, particularly in TB and HIV co-infected children. Lack of disclosure [3], poor ART adherence [16], drug regimens, opportunistic infections (OIs) [3, 5, 9, 16], and short follow-up period [3] were strongly associated with the occurrence of treatment failure among TB and HIV co-infected children in the era of previous studies.

Drug resistance and subsequent treatment failure among HIV-infected children are usually encountered in a resource-limited setting. Even though a lot of efforts have been made in resource-limited settings such as Ethiopia to reduce the incidence of treatment failure, it remains high and happens for a short period, primarily in TB/HIV co-infected children.

Even though first-line treatment failure is higher and has different types of adverse effects or associated complications in a resource-limited setting, there is prior evidence in general and in the study area in particular. Hence, this study was aimed at determining the half-life time prediction of first-line antiretroviral treatment failure and its risk factors among TB and HIV co-infected children in Northwest Ethiopia.

## Methods

### Study design, period, and setting

A retrospective follow-up study was conducted among TB and HIV co-infected children from September 2010 to December 2020 in two compressive specialized hospitals in the Northwest Amhara region of Ethiopia. In particular, these hospitals, namely Debre Tabor and University of Gondar compressive specialized hospitals, are located 103 and 166 km from Bahir Dair City, the capital of Amhara regional state, and 748 km from Addis Ababa, the capital city of Ethiopia, respectively. The hospitals serve more than 7 million people in the region and those nearest to it. The ART clinic is one of the services provided since 2005 by G. C for both adults and children living with HIV. There were 2089 children on ART (15 years) in both hospitals during the study.

#### Study participants

The study included all TB and HIV co-infected children at Debre Tabor and University of Gondar compressive specialized hospitals from September 2010 to December 2020 whose data was incomplete (which means medical records with an unknown status of treatment failure) were excluded from the study.

#### Sample size determination

The sample size was calculated by using Log-rank survival data analysis of the two-population proportion formula by considering the following assumptions: 95% confidence level (Cl), 80% optimum statistical power, and taking type one error of 5%. By considering the previous study conducted in Addis Ababa, Ethiopia [2], and taking age 36 months as the exposed group denoted by q1 (0.78) and age > 60 months as the non-exposed group denoted by q0 (0.90), the total sample size after adding 10% incomplete medical records was 334. However, all TB and HIV co-infected children eligible for this study from September 2010 to December 2020 in Debre Tabor and University of Gondar compressive specialized hospitals were 246. Therefore, we include all study participants for this study.

### Operational definitions

The Half-life time is defined as the time required for half of the study participants to develop the event (i.e., treatment failure).

Treatment failure: The event or outcome variable of the study was treatment failure, including clinical and/or immunological and/or virological failure [16]. Clinical failure: A new or recurrent clinical event indicating advanced or severe immune deficiency (WHO clinical stages 3 and 4) after 6 months of effective treatment [3]. A persistent (at least two CD4 measurements) cluster of differentiation4 (CD4) levels below 200 cells/mm in children under the age of five, and CD4 levels below 100 cells/mm in children over the age of five [3, 16]. Virological failure: the viral load was above 1000 copies/mL based on two consecutive viral load measurements in 3 months [16]. Censored: A study participant without treatment failure (participants such as those who lost follow-up, died, transferred out, or were still on first-line ART at the end of follow-up).

#### Data collection tools and procedures

Data were collected from the medical records of the children by using data extraction. The standard data extraction tool was adapted from the paediatric HIV care guidelines of the Federal Ministry of Health of Ethiopia [17]. Four BSc nurse practitioners collected the data, who were supervised by two MSc in paediatric and child health nurse practitioners. The supervisor checked the completeness of the data every day. Training was given for data collectors and supervisors. In addition, a pre-test was conducted with 5% of the sample size to check the completeness of the medical records.

#### Data processing and analysis

The data were entered into Epi Data version 4.2.2 and exported to STATA version 14.0 for analysis. Descriptive statistics were shown through graphs, frequency, and proportion. A Kaplan-Meier plot was used to estimate the half-life time of the children developing treatment failure. In addition, the Log-rank test was used to see the degree of discrepancy between the predictor variable and the actual one. The Cox proportional hazard model was checked through the Schoenfeld residual test (global test = 49.7) and a parallel graphical proportional hazard assumption.

The bivariable Cox-proportional hazard regression models were fitted for each predictor variable after the required assumption was fulfilled. Furthermore, variables with a *p*-value < 0.25 in bivariate analysis were fitted into a multivariable Cox-proportional hazards regression model, and variables with a *P*-value < 0.05 in multivariate analysis were used to declare a significant association for developing first-line treatment failure.

## Results

### Socio-demographic characteristics

Of the 246 TB and HIV co-infected children, 239 were included in the analysis, yielding a response rate of 97.2%. Of the total number of children, nearly half (54.39%) were males. The children were 7.78 (3.34 standard deviation (SD) years old on average. One hundred twenty-six (52.72%) of the children were aged 5–9 years. The majority (83.26%) of the children lived in urban areas. In addition, one hundred fifty (62.76%) of children knew their HIV status (Table 1).

**Table 1:** Socio-demographic characteristics among TB and HIV Co-infected Children in Northwest Ethiopia from September 2010-December 2020

Variable	Frequency (*n* = 239)	Percentage (*n* = 100%)
Age		
< 5 years	59	24.69
5-9 years	126	52.72
≥ 10 years	54	22.59
Sex		
Male	130	54.39
Female	109	45.61
Residence		
Urban	199	83.26
Rural	40	16.74
Disclosure status		
Non disclosed	89	37.24
Disclosed	150	62.76

### Clinical characteristics

At the end of the follow-up, 42 (17.6%) of the children died during the follow-up study. Besides, 57 (23.8%) of children developed first-line antiretroviral treatment failure. Of these, 32 (13.39%), 16 (6.69%), and 9 (3.77%) were clinical failures, immunological failures, and virological failures, respectively. A total of 71 (29.71%), 51 (21.34%), and 53 (22.18%) of children had CD4 counts below the threshold level, hemoglobin (HGB) 10 mg/dl, and WHO clinical stage IV, respectively. Regarding nutritional status, 61 (25.52%) and 103 (43.10%) of children had severe acute malnutrition and stunting, respectively. In addition, regarding prophylaxis therapy, 196 (82.11%) and 73 (30.54%) of children were Cotrimoxazole preventive therapy (CPT) and Isoniazid preventive therapy (IPT) users, respectively. More than one-third (30.13%) of children experienced ART drug side effects. Initially, 144 children (72.80%) were introduced to the Neverpin (NVP) regimen. Moreover, more than two-thirds (74.90%) and 163 (68.20%) of children had a good level of adherence to ART and initiated ART before a test and treat (2014) strategy was launched or implemented (Table 2).

**Table 2:** Clinical, characteristics among TB and HIV Co-infected Children in Northwest Ethiopia from September 2010-December 2020

Variable	Frequency (*n* = 239)	Percentage (*n* = 100%)
WHO clinical staging		
Stage III	186	77.82
Stage IV	53	22.18
CD4 count or CD4%		
Below threshold level	71	29.71
Above threshold level	168	70.29
Hemoglobin level		
*≤* 10 g/dl	51	21.34
> 10 g/dl	188	78.66
Weight/Height		
Normal	178	74.48
SAM	61	25.52
Height/age		
Normal	136	56.90
Stunting	103	43.10
CPT		
Yes	196	82.01
No	43	17.99
IPT		
Yes	73	30.54
No	166	69.46
Regimen given		
NVP	174	72.80
EFV	55	23.01
LVP/r	10	4.18
Drug side effect		
Yes	72	30.13
No	167	69.87
ART adherence		
Good	179	74.90
Fair /Poor	60	25.10
Year of initiation		
After test and teat (> 2014)	88	36.82
Before test and treat (< 2014)	151	63.18
Duration of follow-up in months		
*≤* 34 months	76	31.80
> 34 months	163	68.20
Final outcome		
No	182	76.15
Clinical failure	32	13.39
immunological	16	6.69
virological failure	9	3.77
Survival status		
live	197	82.4
died	42	17.6

*ART* Antiretroviral Therapy, *CD4* cluster of differentiation, *CPT* Cotrimoxazole preventive therapy, *IPT* Isoniazid preventive therapy, *WHO* World Health Organization, *EFV* Efavirenz, *LPV/r* Lopinavir, *NVP* Nevirapine, *SAM* Severe acute malnutrition

### The half-life time to develop first-line antiretroviral treatment failure

**Fig. 1:**
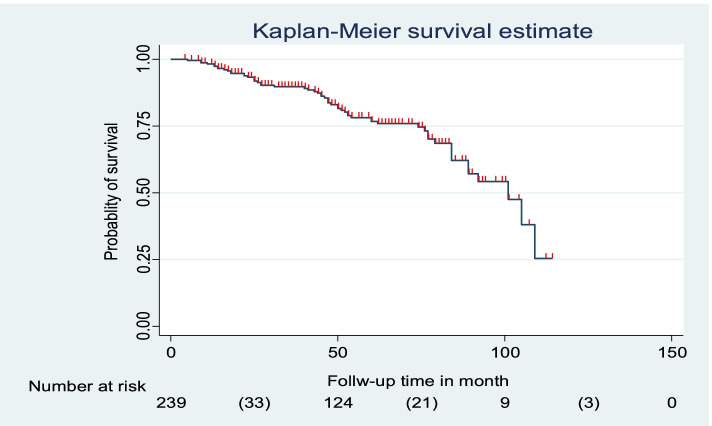
Kaplan mere curve shows the half-life time of developing First-line antiretroviral treatment failure among TB and HIV Co-infected Children in Northwest Ethiopia from September 2010-December 2020

The mean follows up period was 51.6 (±27.1 SD) months with a total 1027.8 years follow uptime. The overall half-life time of developing first-line treatment failure was found to be 101 months. The incidence rate and proportion of developing first-line treatment failure were 5.5 per 100 PPY [CI: 3.7, 6.9] 100 PPY and 23.8% (CI; 18.8, 29.7) respectively (Fig. 1).

### Bivariable and multivariable cox proportional hazard analysis

Firstly, all variables fitted into the bivariable Cox proportional hazard model. Secondly, variables including age, residence, WHO clinical staging, CD4 count or CD4%, hemoglobin level, weight/height, CPT, drug side effects, level of adherence to ART, and year of initiation were significant variables in the bivariable study for the multivariable with a *P* value less than 0.25. Finally, variables including Hgb 10 mg/dl, severe acute malnutrition, WHO stage IV, and CPT non-users were found to be significant variables in a multivariable study for risk factors for developing first-line treatment failure.

The risk of developing first-line treatment failure was 3.2 times higher in children with Hgb 10 mg/dl than in children without [AHR: 3.2 (95% CI: 1.30, 7.73)]. Likewise, the hazard of developing first-line treatment failure was 3.8 times higher among children who had severe acute malnutrition as compared with children without severe acute malnutrition [AHR: 3.8 (95% CI: 1.51, 79.65)].

**Fig. 2:**
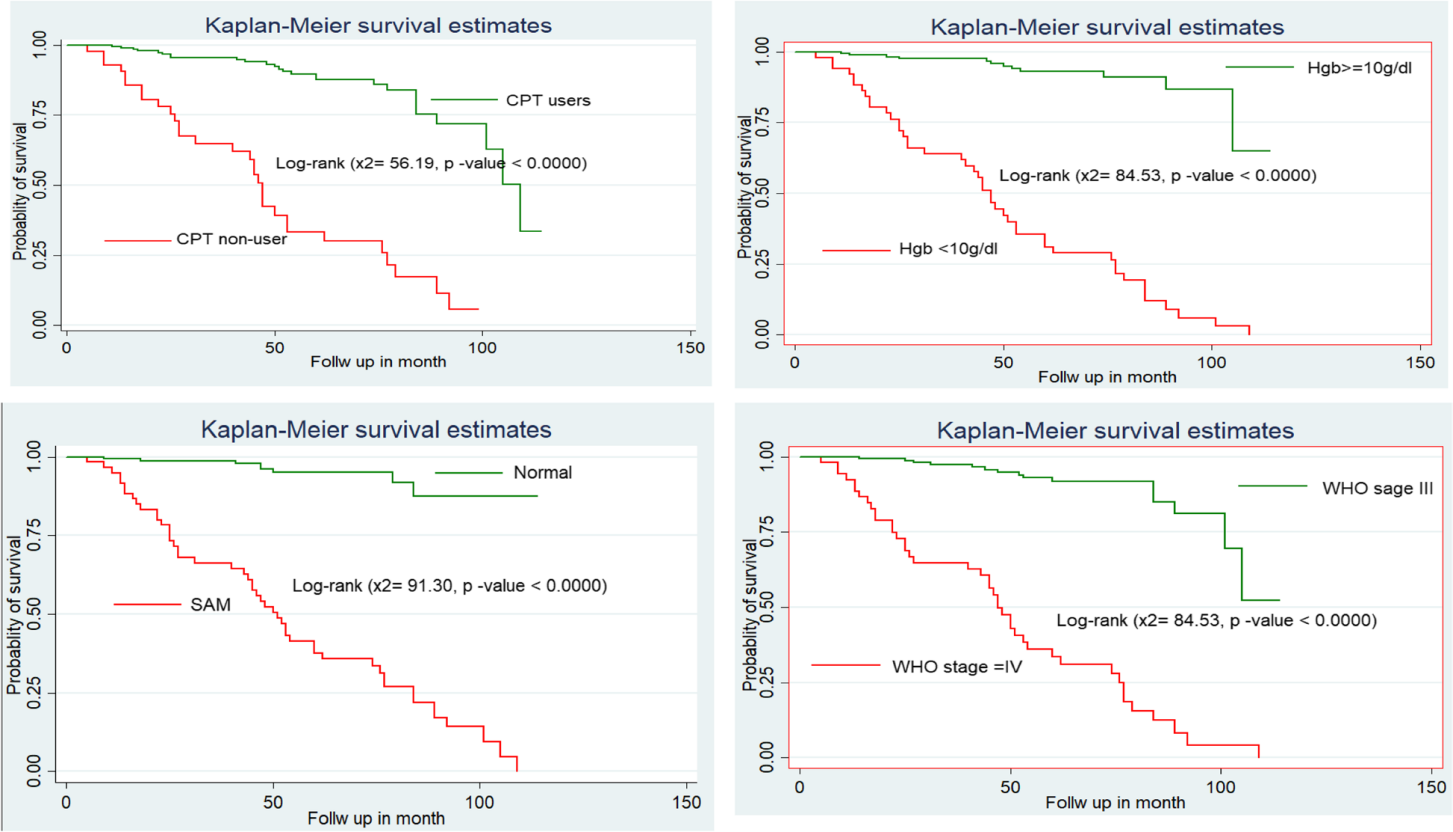
Kaplan mere curve shows the predictor variable of developing First-line antiretroviral treatment failure among TB and HIV Co-infected Children in Northwest Ethiopia from September 2010-December 2020

The hazard of developing first-line treatment failure was 2.3 times higher among children who were CPT non-users as compared with children who were CPT users [AHR: 2.3 (95% CI: 1.14, 4.47)]. In addition, the hazard of developing first-line treatment failure was 2.4 times higher among children who had WHO stage IV as compared with children who had WHO stage III [AHR: 2.4 (95% CI: 1.15, 4.93)] (Table 3). Besides, the Kaplan-Merely curve and Log-rank test were estimated to see the degree of discrepancy between the predictor variable and (Fig. 2).

**Table 3:** Cox-proportional hazard analysis of Predictors of first-line ART failure among TB and HIV Co-infected Children in Northwest Ethiopia

Characteristics	Treatment failure		HR (95% CI)		
	Censored (182)	Event (57)	CHR	AHR	*P*-value
Age					
< 5 years	43	16	1.8 (0.84,3.94) *	1.7 (0.72,3.94)	0.23
5-9 years	96	30	1.1 (0.56,2.23) *	0.9 (0.40,1.89)	0.77
≥ 10 years	43	11	1	1	
Sex					
Male	99	31	1.2 (0.68,1.96)	_	
Female	83	26	1	_	
Residence					
Urban	155	44	1	1	
Rural	27	13	2.0 (1.07,3.73) *	1.6 (0.75,3.27)	0.23
Disclosure status					
Non disclosed	65	24	1.3 (0.75,2.15)	_	
Disclosed	117	33	1	_	
WHO clinical staging					
Stage III	171	15	1	1	
Stage IV	11	42	13 (7.18,23.5) *	2.4 (1.15,4.93) **	0.020
CD4 count or % level					
Below threshold	26	45	9.4 (4.98,17.8) *	1.7 (0.75,3.78)	0.206
Above threshold	156	12	1	1	
Hemoglobin level					
*≤* 10 g/dl	15	36	15 (8.42,30.2) *	3.2 (1.30,7.73) **	0.012
> 10 g/dl	167	21	1	1	
Weight/Height					
Normal	163	15	1	1	
SAM	19	42	17 (8.43,37.7) *	3.8 (1.51,9.65) **	0.005
Height/age					
Normal	106	30	1	_	
Stunting	76	27	1.0 (0.62,1.78)	_	
CPT					
Yes	170	26	1		
No	12	31	8.6 (4.97,14.7) *	2.3 (1.14,4.47) **	0.020
IPT					
Yes	60	13	1	_	
No	122	44	1.4 (0.75,2.58)	_	
Regimen given					
NVP	138	36	0.6 (0.211,1.76)	_	
EFV	38	17	0.9 (0.28,2.62)	_	
LVP/r	6	4	1	_	
Drug side effect					
Yes	54	18	1.1 (0.63,1.95) *	0.8 (0.38,1.72)	0.574
No	128	39	1	1	
ART adherence					
Good	143	36	1		
Fair /Poor	39	21	2.6 (1.50,4.45) *	2.0 (0.94,4.40)	0.070
Year of initiation					
After test and treat	79	9	1		
Before test and treat	103	48	2.2 (1.10,4.45) *	0.4 (0.16,1.10)	0.069
Duration of follow-up					
*≤* 34 months	54	22	1.2 (0.9-2.9)	_	
> 34 months	128	35	1	_	

* variables significant in the bivariate at *p*-value ≤ 0.25 at 95% CI

** variables significant in multivariable at *p*-value < 0.05 at 95% CI

*AHR* Adjusted Hazard Ratio; *CHR* Crude Hazard Ratio, *CI* confidence interval, *SAM* Severe acute malnutrition

## Discussion

The overall half-life for developing first-line treatment failure was found to be 101 months. In addition, the treatment failure rate and proportion of developing first-line treatment failure were 5.5 per 100 PPY [CI: 3.7, 6.9] 100 PPY and 23.8% (CI: 18.8, 29.7)**,** respectively.

The rate of treatment failure in this study was consistent with a study conducted in Mozambique [9]. However, the rate of treatment failure in this study was lower than in the studies conducted in Uganda [18] and Nigeria [19].

On the other hand, the rate of treatment failure in this study was higher than in the studies conducted in Ethiopia [2, 3, 16, 20], India [21], and Ireland [22].

This discrepancy might be primarily due to a difference in the target population, since in this study the target adult population were TB and HIV co-infected children, whereas in other settings it was HIV-infected children. This might have increased the rate of treatment failure in this study. Secondly, this variation might be due to the difference in a study setting, study period, cultural difference, and quality of care or service.

In this study, children who had Hgb 10 mg/dl had an increased risk of first-line treatment failure nearly three-fold [AHR: 3.2 (95% CI: 1.30, 7.73)] than those who had Hgb > 10 mg/dl. Children with anemia have a common problem of decreasing quality of life, functional capacity, and survival. Additionally, anemia has been widely reported to predict a poorer prognosis among children with HIV and TB by increasing the risk of malnutrition, CD4 count depletion, and opportunistic infection, which can result in treatment failure of antiretroviral therapy [23, 24].

Children with severe acute malnutrition were nearly three times more likely to fail first-line treatment [AHR: 3.8 (95% CI: 1.51, 79.65%)] than children without severe acute malnutrition. This could be because children with severe acute malnutrition are more likely to develop infectious co-morbidities such as pneumonia, tuberculosis, gastroenteritis, candidiasis, and other complications such as treatment failure [25, 26].

Children who were CPT non-users had an increased risk of first-line treatment failure nearly two-fold [AHR: 2.3 (95% CI: 1.14, 4.47)] than children who were CPT users. In fact, cotrimoxazole can prevent the development of some of the most serious opportunistic infections in patients with human immunodeficiency virus, primarily by decreasing rates of malaria, pneumonia, and diarrhoea, which can decrease the incidence of treatment failure phenomena and can improve the survival of children [27–29].

Children who had WHO stage IV had an increased risk of first-line treatment failure nearly two-fold [AHR: 2.4 (95% CI: 1.15, 4.93)] than children who had WHO stage III. This finding is consistent with other studies [9, 16, 30, 31]. Actually, advanced opportunistic infection remains the major driver of HIV-associated morbidity and mortality in HIV-infected children by deteriorating the immune system and by worsening the side effects of ART drugs, which can cause treatment failure later on [32, 33].

As a result, it is critical to pay more attention to children with hemoglobin levels of 10 mg/dl, severe acute malnutrition, and WHO stage IV in terms of monitoring and evaluating them on a regular basis. Furthermore, strategies for improving cotrimoxazole preventive therapy for all TB and HIV co-infected children to reduce the occurrence of first-line treatment failure and its associated complications were endorsed.

This study has some limitations. Firstly, since this study was a retrospective cohort, some variables were not accessible in the medical records and, therefore, were not included in this study. Secondly, those study participants whose charts did not have an outcome variable were not included in the study, which may under estimate the rate or proportion of treatment failure.

## Conclusion

In this study, the half-life time to develop first-line treatment failure was found to be very low. In addition, the incidence was found to be very high. The presence of hemoglobin 10 mg/dl, severe acute malnutrition, World Health Organization stage, and non-use of cotrimoxazole prophylaxis were discovered to be risk factors for treatment failure. Further prospective cohort and qualitative studies should be conducted to improve the quality of care in paediatric ART clinics to reduce the incidence or burden of first line treatment failure among TB and HIV co-infected children.

## Data Availability

The datasets used and/or analysed during the current study are available from the corresponding author on reasonable request.

## Abbreviations

AHR: Adjusted Hazard Ratio
ART: Antiretroviral Therapy
CD4: Cluster of differentiation
CHR: Crude Hazard Ratio
CI: Confidence interval
CPT: Cotrimoxazole preventive therapy
EFV: Efavirenz
HIV: Human Immune Deficiency Virus
HR: Hazard Ratio
IPT: Isoniazid preventive therapy
LPV/r: Lopinavir
NVP: Neverpin
OI: Opportunistic Infection
TB: Tuberculosis
WHO: World Health Organization
